# A Poor-Quality Generic Drug for the Treatment of Visceral Leishmaniasis: A Case Report and Appeal

**DOI:** 10.1371/journal.pntd.0001544

**Published:** 2012-05-29

**Authors:** Thomas P. C. Dorlo, Teunis A. Eggelte, Gerard J. Schoone, Peter J. de Vries, Jos H. Beijnen

**Affiliations:** 1 Division of Infectious Diseases, Academic Medical Center, University of Amsterdam, Amsterdam, The Netherlands; 2 Department of Pharmacy & Pharmacology, Slotervaart Hospital / the Netherlands Cancer Institute, Amsterdam, The Netherlands; 3 Department of Parasitology, KIT Biomedical Research, Amsterdam, The Netherlands; Institute of Tropical Medicine, Belgium

A generic miltefosine pharmaceutical product containing no active pharmaceutical ingredient for the treatment of visceral leishmaniasis emerged in Bangladesh for use in the national elimination programme. Poor-quality drugs for the treatment of this fatal neglected tropical disease are life-threatening for the vulnerable patients using them but also have a devastating impact on public health and elimination programmes targeting this disease. National drug regulators should take responsibility and ensure without any concessions that procured drugs for neglected tropical diseases, either innovator or generic, adhere to international standards for drug quality and safety.

## Introduction

Proper chemotherapy is pivotal in the management of visceral leishmaniasis (VL, also known as kala-azar); without an effective treatment this neglected parasitic disease is inevitably fatal [1]. Nevertheless, the few new and safer but more expensive treatment options that were developed in the past decade (i.e., liposomal amphotericin B and miltefosine) remain largely out of reach of the affected rural population who are most in need, mainly the poorest of the poor [2]–[4]. Miltefosine, an alkylphosphocholine drug, is an essential drug in the management of VL as it is the first effective oral treatment option with a reasonable safety profile [5]. Oral miltefosine allows the treatment of VL patients without an extended period of hospital admission and thus puts fewer demands on both patients and health services [6], [7]. Miltefosine is currently preferred for implementation in national VL elimination programmes [8], although the burden of high treatment costs incites the exploration of possibilities for a generic miltefosine product [9]. Unfortunately, the precarious position of VL patients was recently jeopardized as patients in Bangladesh were confronted with a new threat: the emergence of a new miltefosine product containing no active pharmaceutical ingredient [10], [11].

## Case Description

**Figure 1:**
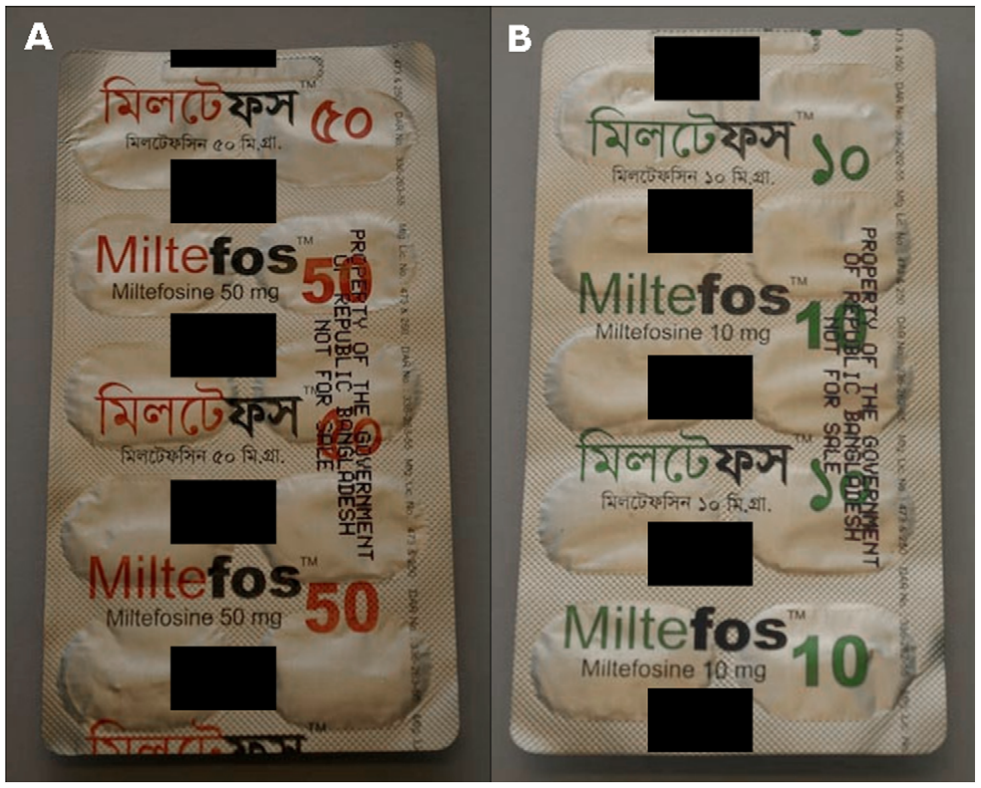
“Miltefos” blister packs. Backs of blister package “Miltefos, Miltefosine 50 mg” (A) and “Miltefos, Miltefosine 10 mg” (B), respectively. The name and logo of the manufacturer have been obscured.

Together with Nepal and India, the government of Bangladesh has committed to eliminate VL by 2015, supported by the World Health Organization (WHO) [8], [12]. Interventions in this VL elimination programme comprise active case surveillance and implementation of vector control management strategies, but also improvement of the availability of appropriate drugs [13]. Oral miltefosine was recommended for this strategy and was therefore registered in Bangladesh [14]. Nevertheless, problems relating to its procurement and supply prohibited accessibility of this treatment and required a less costly alternative. Local procurement of miltefosine was therefore sought and a generic product supposedly containing miltefosine named “Miltefos” was manufactured by a local company for use in the Bangladeshi national elimination programme for VL. In early 2008, “Miltefos” was implemented as first-line therapy for VL in Bangladesh [8]. Although official numbers remain absent, reports from the field indicated abnormal “poor responses in hundreds of patients” after the use of “Miltefos” [8], [10], thereby clearly contradicting high historic efficacy rates (∼95%) of miltefosine in VL in nearby Indian and Nepalese provinces [15]. Therefore bioequivalence studies were planned to compare the local generic “Miltefos” product to the innovator “Impavido” product (Paladin Labs); however, the validity of the underlying assumption of pharmaceutical equivalence had to be established first. For this reason drug samples were sent from Bangladesh to our lab to analyze them for their miltefosine content. To our best knowledge, only two different “Miltefos” batches were produced and distributed in Bangladesh and representative drug samples from both these batches of “Miltefos” with respective label claims of “10 mg miltefosine” and “50 mg miltefosine” (Figure 1) were analyzed. A platform of analytical techniques was developed of which the methodology is described in more detail elsewhere [11]. A high-performance liquid chromatography coupled to tandem mass spectrometry (LC-MS/MS) method validated for the detection of miltefosine with a lower limit of quantitation of 4 ng/ml in human plasma immediately revealed that no miltefosine could be identified in methanol extracts of any of the “Miltefos” capsules, while this method allowed the irrefutable identification and quantification of miltefosine in the “Impavido” capsules [16]. This absence of miltefosine was confirmed in the crude “Miltefos” capsule contents by Fourier transform infrared spectroscopy and a new, simple, and rapid colorimetric test for miltefosine with a lower limit of detection of at least 12.5 µg/ml [11]. Near-infrared spectroscopy even allowed us to confirm this finding without opening the capsules. Further tests using mass spectrometry could not identify any other compound, including any possible degradation products of miltefosine, in the “Miltefos” capsule contents besides the common excipients lactose and microcrystalline cellulose [11].

To confirm these findings and to ensure that the pharmaceutical product under investigation was the same product that was being used by Bangladeshi VL patients, a second representative sample of “Miltefos” specimens was collected directly in the field in Bangladesh at a health centre where VL patients were under treatment with “Miltefos” at that time. Furthermore, whole blood samples from five VL patients who were still under treatment with “Miltefos” and the exact blisters that had been used to treat these patients were collected simultaneously. Both these blood and drug specimens were transported to our lab in sealed and signed envelopes to ensure the integrity of the parcel in transit. Case record forms and signed statements by the doctors of these patients indicating the origin and authenticity of the samples were included. The same analytical techniques as mentioned above reconfirmed the previous results: no miltefosine could be identified in these “Miltefos” batches, nor any other active pharmaceutical ingredient. Storage conditions in Bangladesh or during transport are not expected to have been of any influence, given miltefosine's excellent stability profile, also in humid and hot conditions [17]. Moreover, no miltefosine could be detected in the whole blood samples of the patients to whom these “Miltefos” capsules were administered. All these patients were already several days on “Miltefos” treatment and should have accumulated substantial miltefosine blood concentrations because of the extremely slow elimination of miltefosine from the body [18]. A real-time reverse-transcriptase PCR (qRT-PCR) targeting the *Leishmania* 18S ribosomal RNA performed on blood of these patients confirmed that they were suffering from VL, indicating the authenticity of the received samples [19]. Following these findings, clinical care of the patients was changed to treatment with intravenous sodium antimony gluconate. Eventually, “Miltefos” was removed from all patient care regimens throughout Bangladesh and intravenous sodium antimony gluconate was re-introduced as first-line therapy for VL in Bangladesh [8].

## Discussion

This case demonstrates that despite existing international regulations for quality assurance of medicines, poor-quality drugs can be distributed through a nationwide treatment programme in a resource-poor country and reach patients suffering from a fatal neglected tropical disease whose survival depends on good-quality drugs. The control of anthroponotic VL in South Asia relies most importantly on early case detection and effective treatment, thereby reducing the human reservoir of disease [20], [21]. The use of substandard or counterfeit drugs containing no or a subtherapeutic amount of active pharmaceutical ingredient not only severely jeopardizes the individual health of patients but also these control efforts for VL. This emphasizes the need for prioritizing the quality of anti-infective medicines that are being used in resource-poor countries to treat neglected tropical diseases and the development of simple and rapid methods to assess drug quality [22]–[24].

The poor-quality generic miltefosine product that we investigated can be considered as a substandard product according to WHO definitions, since it never contained any active pharmaceutical ingredient [25]–[27]. However, this product cannot directly be assumed to be a counterfeit as well, since a fraudulent motive for the mislabelling of this product cannot be inferred from our scientific investigation [28]. For VL, at least three previous cases of poor-quality drugs have been described in India, Nepal, and Sudan, all concerning antimonials and resulting in unacceptable toxicity and even death [29]–[31]. Poor-quality medicines, both substandard and counterfeit, constitute a major burden on the public health in resource-poor countries [10], [22], [32], [33]. Inadequate local drug regulation and law enforcement combined with poor compliance of local pharmaceutical industry with good manufacturing practices (GMP) can lead to high rates of substandard drug production in resource-poor countries, but also to a higher degree of deliberate counterfeiting activities [22], [34]. Antimalarials have most extensively been reported as a victim of counterfeiting in resource-poor countries, with a focus on artesunate products containing no or only a subtherapeutic amount of active pharmaceutical ingredient [35]–[37]. Provision of free or inexpensive antimalarials has been mentioned as one of the possible solutions to this widespread problem, removing the financial incentive for counterfeiters [32]. It has also been suggested that the quality of drugs would be warranted if distributed “through official institutions” [4]. However, this case clearly underlines that provision of free anti-infectives—even through official institutions—does not necessarily imply that the problem of poor-quality anti-infectives in resource-poor countries is resolved and strict monitoring of drug quality by the funding organizations or responsible government bodies is therefore key in any drug provision programme.

Without ignoring the ongoing debate on the various definitions of “substandard” and “counterfeit” [22]–[24], [27], [34], [38], the emphasis in tackling poor-quality drugs should be on the safety of patients and public health, especially in resource-poor countries and certainly concerning the treatment of life-threatening diseases. Shifting the focus from intellectual property or trade issues towards public health and patients to define and combat poor-quality drugs is therefore urgently needed [38]. Quality assurance of pharmaceutical products should be guaranteed by appropriate (government) bodies that have the specific mandate to protect individuals and public health, and should not be the responsibility of vulnerable individual patients themselves [34], [39]. In resource-poor countries it remains cumbersome to provide this protection, partly due to an urgent lack of both drug regulatory capacity and regional analysis laboratories for quality control of medicines. Previously, it has been shown that GMP compliant facilities can have parallel productions with lower standards for poorly regulated countries [34]. Quality assurance should therefore specifically extend to assessment of each manufacturing site and each product dossier according to the rigorous criteria set by the WHO for each individual drug that is being procured [40]. Several programmes have been initiated by the WHO (most notably the WHO Prequalification Programme) but separately also by other organizations (e.g., Médecins Sans Frontières) to facilitate qualification of drugs and access to technical expertise on this topic [34], [41]. Unfortunately, the WHO Prequalification Programme selectively focuses on good-quality medicinal products relating to HIV/AIDS, tuberculosis, malaria, influenza, and reproductive health and has thus far ignored medicines against neglected diseases, although the availability of general technical expertise could help support regulatory authorities with limited capacities. This case indicates that it may be needed to extend the WHO Prequalification Programme to drugs for VL and other neglected diseases.

Availability and accessibility of regulatory information and technical expertise such as methods of analysis and identification for drugs for neglected diseases need to be prioritized. Currently, no monograph on miltefosine has been included in any of the major international pharmacopoeias, although the drug has been added to the WHO Model List of Essential Medicines and has received approval in Germany, which has a stringent regulatory authority [42], [43]. International pharmacopoeias should cease to neglect drugs for neglected diseases to facilitate the quality control of these medicines and the production of generic pharmaceutical products. Furthermore, no formal approval of miltefosine or “Miltefos” could be traced in the publicly available information of the Bangladeshi drug regulatory authorities [44], despite WHO documents mentioning the registration of miltefosine in Bangladesh [14]. Full transparency of the current regulatory status of medicinal products is simply a prerequisite for the safe and effective use of drugs for neglected diseases, certainly in the resource-poor countries that are most affected.

Nevertheless, all these outstanding efforts can only be effective when national or regional drug regulators and procurement programmes in resource-poor countries are aware of the devastating impact of poor-quality drugs and decide to make use of all the (technical) resources that could be provided to them by international agencies. It must be emphasized that these recommendations certainly do not reject a priori the production or procurement of generic pharmaceutical products as a cheaper alternative for the treatment of neglected tropical diseases; however, no concessions should be tolerated in terms of assurance of quality and safety of these medicines, either innovator or generic.

## Conclusion

VL patients belong to the poorest quintile of the population of resource-poor countries [3], which make them highly dependent on drug donations or drug provision by national elimination programmes for the treatment of their fatal disease. These extremely vulnerable patients deserve to be protected by national or regional drug regulators who should take responsibility by implementing the necessary precautions to prevent repetition of this poor-quality drug case. All procured VL drugs either used in the respective national VL elimination programmes, available over the counter, or used in clinical trials need to adhere to the WHO standards for quality and safety, irrespective of the country of origin of these drugs, whether they are innovator or generic medicines and whether they are intended to be used in resource-rich or resource–poor countries. Only under this condition can VL patients trust in a safe treatment, and national elimination programmes might have an impact on this typically neglected disease.

### Ethics Statement

Written informed patient consent was obtained for the blood samples. Institutional review board approval was not required, since patients were not treated in the context of a study and the described procedures on the blood samples were performed to ensure that these patients received adequate treatment.
